# Correlation between ERK1 and STAT3 expression and chemoresistance in patients with conventional osteosarcoma

**DOI:** 10.1186/1471-2407-14-606

**Published:** 2014-08-20

**Authors:** Sébastien Salas, Carine Jiguet-Jiglaire, Loic Campion, Catherine Bartoli, Frédéric Frassineti, Jean-Laurent Deville, André Maues De Paula, Fabien Forest, Pascal Jézéquel, Jean-Claude Gentet, Corinne Bouvier

**Affiliations:** Aix Marseille Univ, CRO2, 13284 Marseille, France; INSERM, U911, 13005 Marseille, France; APHM, Timone Hospital, Department of Medicine, Division of adult oncology, 13005 Marseille, France; Integrated Center for Oncology, Biostatistics Unit, Nantes, France; Department of Pathology, APHM, Timone Hospital, 13005 Marseille, France; Department of Biology, Integrated Centre of Oncology, Nantes, France; INSERM U892, IRT-UN, Nantes, France; Department of Medicine, Division of Pediatric Oncology, APHM, Timone Hospital, 13005 Marseille, France

**Keywords:** Conventional osteosarcomas, Predictive factors, Chemotherapy response, *STAT3*, *ERK1*

## Abstract

**Background:**

The standard therapy regimen of conventional osteosarcoma includes neoadjuvant chemotherapy followed by surgical resection and postoperative chemotherapy. The percentage of necrotic tissue following induction chemotherapy is assessed by using the Huvos grading system, which classifies patients as “poor responders” (PR) and “good responders” (GR). The aim of this study was to identify molecular markers expressed differentially between good and poor responders to neoadjuvant chemotherapy in order to predict the response to chemotherapy in conventional osteosarcomas before beginning treatment.

**Methods:**

Suppression Substractive Hybridization (SSH) was performed by using cDNA from frozen biopsy specimens. Expression of selected relevant genes identified by SSH was validated by using QRT-PCR. Immunohistochemistry (IHC) on tissue microarray (TMA) sections of 52 biopsies was performed to investigate protein expression in an independent cohort.

**Results:**

*ERK1* and *STAT3* mRNA level were significantly different between PR and GR in an independent cohort. Phosphorylated STAT3 and ERK1 expressions by IHC on TMA were correlated with poor response to chemotherapy.

**Conclusions:**

Our results suggest that *ERK1* and *STAT3* expression are good predictive markers for chemotherapy response and that inhibitors might be used in combination with common chemotherapeutic drugs in conventional osteosarcomas.

## Background

Osteosarcoma, the most common type of primary bone cancer, is a rare disease. Approximately 900 new cases of osteosarcoma are diagnosed each year in the United States (http://www.cancer.org/docroot/home/index.asp) and 200 in France, including 150 in children (http://www.fnclcc.fr/sor/SSP/CancersEnfant/PeauTissusSoutien/Osteosarcome). Adjuvant and neoadjuvant chemotherapy have significantly improved the long-term survival rate for patients with osteosarcoma [1–3]. Nevertheless, recurrent disease still occurs in about 30–40% of patients and more than 70% of them die of their tumor, despite second-line treatment. The standard therapy regimen of high-grade osteosarcoma includes induction by multiagent chemotherapy followed by surgical resection and postoperative chemotherapy [4]. The percentage of necrotic tissue following induction chemotherapy is classified with the Huvos grading system [5]. Patients with <90% tumor necrosis following induction therapy are classified as “poor responders” (PR) or Huvos grade I/II [6], while more than 90% necrosis corresponds to Huvos grade III and complete necrosis to Huvos grade IV. Good responders (GR) correspond to Huvos grade III/IV. The degree of necrosis remains the only reliable prognostic factor for the patients presenting with localized disease and is used to guide the choice of postoperative chemotherapy. Numerous clinical trials have attempted to increase the disease-free survival rate for poorly responding patients with intensified postoperative therapy. No survival benefit has been convincingly shown through the administration of more intensified therapy to poor responders [3, 7–10]. This suggests that there may be an innate biological difference between good responsive and poor responsive tumors. Previous transcriptomic studies have shown that in chemoresistant tumors, the genes involved in osteoclastogenesis, extracellular matrix remodeling, bone development, tumor progression, drug resistance and angiogenesis are up-regulated [11–17]. However, none of these molecular predictive factors can be used routinely. Therefore, there is a need to establish reliable predictive biomarkers for the response to chemotherapy at the time of diagnosis. The aim of this study was to identify molecular markers expressed differentially between “good” and “poor” responders to neoadjuvant chemotherapy in order to predict the response to chemotherapy in conventional osteosarcoma before beginning treatment, and to elucidate the mechanisms involved in this response. We identified several subsets of novel potential candidate genes. In particular, our data suggest that *ERK1* and *STAT3* expression are involved in the response to chemotherapy and that they could be therapeutic targets.

## Methods

### Patients and tumor specimens

The response to preoperative chemotherapy was assessed on resected specimens according to Rosen’s protocol [4]. To identify differentially expressed genes between “good” and “poor” responders (GR and PR) to chemotherapy, Suppression Subtractive Hybridization (SSH) was performed by using cDNA from frozen biopsy specimens taken for diagnosis prior to treatment. SSH was performed by using 5 samples of GR patients (three males and two females, mean age 14 years) and 4 samples of PR patients (two males and two females, mean age 13.5 years). All patients received preoperative and postoperative chemotherapy derived from the SFOP OS 94 regimen [18]. Clinicopathological characteristics of the patients studied by SSH are presented in Table 1. The two groups were similar in tumor volume, tumor location and histological subtype. Expression of selected relevant genes identified by SSH was validated by using real-time quantitative RT-PCR (QRT-PCR). For QRT-PCR, the same specimens and additional specimens of 22 patients were obtained. The whole cohort consisted of 13 GR and 18 PR. Immunohistochemistry (IHC) was performed on Tissue Microarray (TMA) sections consisting of 52 biopsies of patients with a conventional osteosarcoma. Six of 9 samples used for SSH were used for TMA. 18 of the 31 samples used in QRT-PCR were used for TMA. In total, among the 52 patients in the TMA validation cohort, only 5 received chemotherapy without high-dose MTX. The vast majority of patients (40) were those treated according to protocol OS94 or by neoadjuvant chemotherapy with methotrexate, vepeside and ifosfamide. All samples were obtained after informed consent from patients or their parents when the patients were under the age of 18.

**Table 1:** **Clinicopathological characteristics of the 9 patients studied by SSH**

	Good responders	Poor responders
Number of patients	5	4
Mean age at diagnosis [95% IC] (years)	14 [5-17]	13.5 [13-16]
Sex		
Male	3	2
Female	2	2
Tumor location		
Upper limb	2	1
Lower limb	3	3
Histological subtype		
Osteoblastic	4	3
Osteoblastic and chondroblastic	1	1
Mean tumor size [95% IC] (cm)	12.5 [8-34]	9 [6.7-25]
Mean viable residual tumor cells [95% IC] (%)	2.5 [1-4.5]	25 [17-37]

Research involving the patients have been performed with the approval of Protection of the Person Center: CPP sud Méditerranée 1 ethics committee (authorization number: DC-2008-309) in compliance with the Helsinki Declaration. Samples were from a tumor bank that respects the ethical charter of the French National Cancer Institute (AP-HM Biobank authorization number 2013-1786).

### *RNA*preparation

Total RNA extraction was performed from frozen tumor specimens by using the acid guanidinium isothiocyanate/phenol/chloroform procedure. Before use, RNA samples were treated with 10U ribonuclease-free deoxyribonuclease (Promega, France) at 37°C for 15 min. Tests for purity and quality were performed on a nanodrop spectrophotometer and the Agilent 2100 Bioanalyser RNA LabChip kit (Agilent Technologies, Palo Alto, CA, USA) [19, 20]. Only samples with RNA integrity Number (RIN) > 7 and no evidence of ribosomal degradation were included.

### SMART-Suppression Subtractive Hybridization (SMART: “switching mechanism at 5’ end of the RNA transcript”)

Poly(A) + mRNA were isolated from GR and PR total RNA using an Oligotex mRNA isolation kit (Qiagen, France) and gene expression between these two mRNA populations was compared by SMART-SSH using a Super PCR cDNA Synthesis Kit for cDNA synthesis (Clontech) and a PCR-Select cDNA subtraction kit (Clontech), a principle previously described by Diatchenko et al. [21].

### Cloning and analysis of subtracted clones

Products from the final PCR amplification were cloned into a Topo TA cloning vector (Invitrogen Life Technologies, France) and electro-transferred into One Shot *E. coli*. Differential screening was performed to eliminate false positives. Hybridizations were performed in duplicate according to standard procedures. Specific clones were prepared by using a Qiagen plasmid mini-kit and sequenced (QIAGEN France SAS, Coutaboeuf, France). Nucleic acid homology searches were carried out with the BLAST program at the NCBI, USA.

### Quantitative Reverse Transcription Polymerase Chain Reaction (QRT-PCR)

QRT-PCR was used to accurately detect the changes of expression of selected relevant genes: *ERK1* and *STAT3* gene expression levels and ribosomal 18S RNA as reference sequence. Total RNA (1 μg) DNA-free was reverse-transcribed into cDNA using hexamers (Pharmacia Biotech, Orsay, France) and Superscript II Reverse Transcriptase (Invitrogen Life Technologies, France). Genes of interest and *18S* rRNA were amplified, detected and quantified in real-time by using the Light Cycler Real-Time PCR (Roche Applied Science, Meylan, France). QRT-PCR was performed by using the oligonucleotides and sequence parameters described in Table 2 in a medium containing 1X LightCycler 480 SYBR Green I master mix, 0.25 μM of each primer and 20 ng of cDNA. Each PCR reaction was preceded by one activation cycle of 95°C for 5 min and ended by establishing a melting curve 5 degrees above the oligonucleotide melting temperature.

**Table 2:** **Description of oligonucleotides and sequence parameters for QRT-PCR**

Name gene	Oligo direct	Oligo reverse	PCR conditions	Cycle number	GeneInfo identifier
*18S*	CTACCACATCCAAGGAAGGCA	TTTTTCGTCACTACCTCCCCG	95°C 15 sec	35	124517659
			67°C 30 sec		
*ERK1*	CTAAAGCCCTCCAACCTGCT	CAGCCCACAGACCAGATGT	95°C 15 sec	45	158138506
			60°C 30 sec		
*STAT3*	AAAGTCAGGTTGCTGGTCAAA	TGCCGTTGTTGGATTCTTC	95°C 15 sec	45	76253927
			60°C 30 sec		

### Immunohistochemistry (IHC) on tissue microarray sections (TMA)

Automated immunohistochemistry was performed on slides of TMA paraffin blocks. The 52 tumor specimens were all fixed in 4% formalin. Fleshy tissue was separated from calcified areas to avoid unnecessary decalcification. When necessary, tumor specimens were decalcified in a solution of 22% formic acid. TMA were prepared as previously described [20]. For each sample, three representative sample areas were carefully selected from a hematoxylin–eosin-stained section of a donor block. Core cylinders with a diameter of 1 mm each were punched from three representative areas and deposited onto two separate recipient paraffin blocks by using a specific arraying device (Alphelys). To determine the expression of activated forms of STAT3 and ERK1 proteins, we used anti-phospho-STAT3 (Tyr705) (polyclonal, 9131 from Cell Signaling Technology, dilution 1/20) and anti-phospho-ERK1 (polyclonal, clone 20G11 from Cell Signaling Technology, dilution: 1/100) antibodies. Automated IHC was performed with a Ventana automate (Benchmark XT, Ventana Medical Systems SA, Illkirch, France). Positive external control was a glioblastoma for both pSTAT3 and pERK1. Negative controls were also included and corresponded to omission of primary antibody or irrelevant antibodies of the same isotype. IHC was scored positive when nuclear staining was observed. A semi-quantitative analysis was done for positive specimens without knowledge of clinical data. Percentage of stained cells and staining intensity (weak, moderate, high) were taken into account to obtain the score. Score 0 was attributed to tumors with absence of staining. Score 1 was attributed to tumors with low intensity of staining whatever the number of stained nuclei or to tumors with no more than 25% of nuclei immunostained with moderate intensity. Score 2 corresponded to stained nuclei numbering between 25% and 50% with moderate intensity or to fewer than 25% of stained nuclei with high intensity. Score 3 was defined as either more than 50% of stained nuclei with moderate intensity or more than 25% of stained nuclei with high staining intensity. A mean score was proposed for the three areas of each tumor. Three independent observers evaluated the IHC results blind to clinical data. A consensus score was reached and statistical analysis was performed from the consensus score.

### Data analysis

Relationships between response to chemotherapy (GR vs. PR) and other parameters used were obtained by using non-parametric tests, the Fisher exact test and the Mann-Whitney test when qualitative and continuous respectively. All tests were two-sided. P-value was considered significant when ≤ 5%. SAS System version 9.2 (SAS Institute Inc., Cary, NC) and Stata software (version 10.1 Special Edition, StataCorp, College Station, Texas) were used to perform data analyses.

## Results

### Patients

Clinicopathological characteristics of the patients studied are presented in Table 3.

**Table 3:** **Clinicopathological characteristics of the 52 patients studied by IHC including those studied by SSH and QRT-PCR**

	Whole cohort
Number of patients	52
Age	
Mean age at diagnosis [95% IC]^a^ (years)	17.4 [5;80]
Sex	
Male (%)	34 (65.4)
Female (%)	18 (34.6)
Histologic response	
Good responders	24
Poor responders	28
Histological diagnosis and subtype	
High-grade osteosarcomas of central “conventional” type	52
Osteoblastic (%)	38 (73)
Chondroblastic (%)	5 (9.5)
Telangiectasic (%)	3 (6)
Fibroblastic (%)	2 (4)
Mixed subtype^b^ (%)	4 (7.5)

^a^Confidence Interval, for the whole cohort, to ascertain that the screening cohort is a representative subset of the whole.

^b^Osteoblastic and chondroblastic or fibroblastic.

### Identification of differentially expressed genes by SSH in PR

A subtractive cDNA library of PR was generated. 126 selected clones were sequenced (Table 4). The following genes were selected on the basis of their known roles in tumorigenesis or chemoresistance: *ACTN1, AKT2, ANXA2, CADM1, CDKN2C(P18), FN1, GAL1, HRAS, IGFBP3, LMNA, ERK1* and *STAT3*. Particularly, *STAT3* is a key factor for chemosensitivity in human epithelial ovarian cancer cells and thyroid cancer-derived CD133+ cells [22–24]. Recent studies show that ERKs may also be activated in response to chemotherapeutic drugs, and pERK1/2 played critical roles in drug resistance [25–28]. Thus, these selected genes were tested by QRT-PCR.

**Table 4:** **Identification of genes differentially expressed by SSH in PR**

Gene title	Gene symbol	Chromosomal location
Actin, alpha 1, skeletal muscle	ACTA1	chr1q42.13-q42.2
Actin, beta	ACTB	chr7p15-p12
Actin, gamma 1	ACTG1	chr17q25
Actinin, alpha 1	ACTN1	chr14q24.1-q24.2|14q24|14q22-q24
ADAM metallopeptidase with thrombospondin type 1 motif, 20	ADAMTS20	chr12q12
v-akt murine thymoma viral oncogene homolog 2	AKT2	chr19q13.1-q13.2
Ankyrin repeat domain 11	ANKRD11	chr16q24.3
Annexin A2	ANXA2	chr15q21-q22
AT rich interactive domain 4B (RBP1-like)	ARID4B	chr1q42.1-q43
Actin-related protein 2/3 complex, subunit 2, 34 kDa	ARPC2	chr2q36.1
ATPase family, AAA domain containing 3A	ATAD3A	chr1p36.33
ATP synthase, H + transporting, mitochondrial F0 complex, subunit E///major facilitator superfamily domain containing 7	ATP5I///MFSD7	chr4p16.3
Bromo adjacent homology domain containing 1	BAHD1	chr15q15.1
Breast carcinoma amplified sequence 3	BCAS3	chr17q23
Branched chain aminotransferase 2, mitochondrial	BCAT2	chr19q13
Chromosome 14 open reading frame 112	C14orf112	chr14q24.2
Chromosome 14 open reading frame 2	C14orf2	chr14q32.33
Chromosome 20 open reading frame 194	C20orf194	chr20p13
Cell adhesion molecule 1	CADM1	chr11q23.2
Coiled-coil domain containing 28B	CCDC28B	chr1p35.1
Chaperonin containing TCP1, subunit 8 (theta)	CCT8	chr21q22.11
Cell division cycle 34 homolog (S. cerevisiae)	CDC34	chr19p13.3
Cyclin-dependent kinase inhibitor 2C (p18, inhibits CDK4)	CDKN2C	chr1p32
Carbohydrate (chondroitin 4) sulfotransferase 11	CHST11	chr12q
Creatine kinase, brain	CKB	chr14q32
CDC28 protein kinase regulatory subunit 1B	CKS1B	chr1q21.2
CLPTM1-like	CLPTM1L	chr5pter-p15.3
Cornifelin	CNFN	chr19q13.2
Collagen, type V, alpha 1	COL5A1	chr9q34.2-q34.3
Catechol-O-methyltransferase	COMT	chr22q11.21-q11.23|22q11.21
Cytochrome c oxidase subunit VIa polypeptide 1	COX6A1	chr12q24.2|12q24.2
Cytokine receptor-like factor 1	CRLF1	chr19p12
Chondroitin sulfate glucuronyltransferase	CSGlcA-T	chr7q36.1
Casein kinase 2, alpha prime polypeptide	CSNK2A2	chr16q21
cutA divalent cation tolerance homolog (E. coli)	CUTA	chr6pter-p21.31
dodecenoyl-Coenzyme A delta isomerase (3,2 trans-enoyl-Coenzyme A isomerase)	DCI	chr16p13.3
Dicarbonyl/L-xylulose reductase	DCXR	chr17q25.3
DEAD (Asp-Glu-Ala-As) box polypeptide 19A	DDX19A	chr16q22.1
DEAD (Asp-Glu-Ala-As) box polypeptide 19B///DEAD (Asp-Glu-Ala-As) box polypeptide 19A	DDX19A///DDX19B	chr16q22.1
DEAD (Asp-Glu-Ala-Asp) box polypeptide 39	DDX39	chr19p13.12
Eukaryotic translation elongation factor 1 delta (guanine nucleotide exchange protein)	EEF1D	chr8q24.3
Eukaryotic elongation factor-2 kinase	EEF2K	chr16p12.1
Eukaryotic translation initiation factor 3, subunit H	EIF3H	chr8q24.11
Eukaryotic translation initiation factor 4 gamma, 3	EIF4G3	chr1p36.12
Fas apoptotic inhibitory molecule 3	FAIM3	chr1q32.1
FK506 binding protein 7	FKBP7	chr2q31.2
Kappa-actin	FKSG30	chr2q21.1
Flavin containing monooxygenase 5	FMO5	chr1q21.1
Fibronectin 1	FN1	chr2q34
FERM domain containing 5	FRMD5	chr15q15.3
Golgi SNAP receptor complex member 2	GOSR2	chr17q21
Glypican 1	GPC1	chr2q35-q37
G protein-coupled receptor 108	GPR108	chr19p13.3
Ribosomal protein L23a///similar to ribosomal protein L23A///ribosomal protein L23a-like	hCG_16001///hCG_2001000///RPL23A	chr17q11///chr17q23.2///chr3q26.1
v-Ha-ras Harvey rat sarcoma viral oncogene homolog	HRAS	chr11p15.5
Heparan sulfate proteoglycan 2	HSPG2	chr1p36.1-p34
Insulin-like growth factor 2 mRNA binding protein 3	IGF2BP3	chr7p11
Inositol(myo)-1(or 4)-monophosphatase 2	IMPA2	chr18p11.2
Integrator complex subunit 1	INTS1	chr7p22.3
Importin 11	IPO11	chr5q12.1
Jumonji domain containing 2C	JMJD2C	chr9p24.1
KIAA0999 protein	KIAA0999	chr11q23.3
Laminin, alpha 4	LAMA4	chr6q21
Lectin, galactoside-binding, soluble, 1 (galectin 1)	LGALS1	chr22q13.1
Lamin A/C	LMNA	chr1q21.2-q21.3
Ribosomal protein S16///similar to 40S ribosomal protein S16	LOC441876///RPS16	chr19q13.1///chr1p36.21
Leucine-rich repeat containing 28	LRRC28	chr15q26.3
Microtubule-associated protein 1S	MAP1S	chr19p13.11
Mitogen-activated protein kinase 3	MAPK3 (ERK1)	chr16p11.2
Major facilitator superfamily domain containing 5	MFSD5	chr12q13.13
Mitochondrial ribosomal protein S7	MRPS7	chr17q25
NADH dehydrogenase (ubiquinone) 1 alpha subcomplex, 4, 9 kDa	NDUFA4	chr7p21.3
NADH dehydrogenase (ubiquinone) Fe-S protein 7, 20 kDa (NADH-coenzyme Q reductase)	NDUFS7	chr19p13.3
NADH dehydrogenase (ubiquinone) flavoprotein 1, 51 kDa	NDUFV1	chr11q13
Nuclear factor I/C (CCAAT-binding transcription factor)	NFIC	chr19p13.3
NOL1/NOP2/Sun domain family, member 5	NSUN5	chr7q11.23
NOL1/NOP2/Sun domain family, member 5B	NSUN5B	chr7q11.23
NOL1/NOP2/Sun domain family, member 5C	NSUN5C	chr7q11.23
Nucleoporin 214 kDa	NUP214	chr9q34.1
Nucleoporin 85 kDa	NUP85	chr17q25.1
PDZ domain containing 2	PDZD2	chr5p13.3
Periplakin	PPL	chr16p13.3
Protein phosphatase 1, regulatory (inhibitor) subunit 12B	PPP1R12B	chr1q32.1
Protein phosphatase 2 (formerly 2A), regulatory subunit A, alpha isoform	PPP2R1A	chr19q13.33
Protein kinase C substrate 80 K-H	PRKCSH	chr19p13.2
Protein arginine methyltransferase 2	PRMT2	chr21q22.3
RNA binding protein, autoantigenic (hnRNP-associated with lethal yellow homolog (mouse))	RALY	chr20q11.21-q11.23
RNA binding motif protein 4	RBM4	chr11q13
RNA binding motif protein 4B	RBM4B	chr11q13
RNA binding motif protein 8A	RBM8A	chr1q12
Ribosomal protein L13	RPL13	chr16q24.3|17p11.2
Ribosomal protein L13a	RPL13A	chr19q13.3
Ribosomal protein L19	RPL19	chr17q11.2-q12
Ribosomal protein L23a	RPL23A	chr17q11
Ribosomal protein L31	RPL31	chr2q11.2
Ribosomal protein, large, P1	RPLP1	chr15q22
Ribosomal protein S12	RPS12	chr6q23.2
Ribosomal protein S14	RPS14	chr5q31-q33
Ribosomal protein S17	RPS17	chr15q
Ribosomal protein S21	RPS21	chr20q13.3
Ribosomal protein S27 (metallopanstimulin 1)	RPS27	chr1q21
Ribosomal protein S6	RPS6	chr9p21
RNA pseudouridylate synthase domain containing 4	RPUSD4	chr11q24.2
Ribosomal RNA processing 1 homolog B (S. cerevisiae)	RRP1B	chr21q22.3
Retinoid X receptor, alpha	RXRA	chr9q34.3
Synaptonemal complex protein SC65	SC65	chr17q21.2
Splicing factor, arginine/serine-rich 3	SFRS3	chr6p21
Serine hydroxymethyltransferase 2 (mitochondrial)	SHMT2	chr12q12-q14
SIVA1, apoptosis-inducing factor	SIVA1	chr14q32.33
SIVA1, apoptosis-inducing factor	SIVA1	chr14q32.33
Solute carrier family 16, member 8 (monocarboxylic acid transporter 3)	SLC16A8	chr22q12.3-q13.2
Solute carrier family 20 (phosphate transporter), member 2	SLC20A2	chr8p12-p11
Small nuclear ribonucleoprotein D3 polypeptide 18 kDa	SNRPD3	chr22q11.23
Signal transducer and activator of transcription 3 (acute-phase response factor)	STAT3	chr17q21.31
Serine/threonine kinase 24 (STE20 homolog, yeast)	STK24	chr13q31.2-q32.3
T-cell, immune regulator 1, ATPase, H + transporting, lysosomal V0 subunit A3	TCIRG1	chr11q13.2
Testis-specific kinase 1	TESK1	chr9p13
Thymosin, beta 10	TMSB10	chr2p11.2
Transportin 3	TNPO3	chr7q32.1
Tetraspanin 9	TSPAN9	chr12p13.33-p13.32
Ubiquitin A-52 residue ribosomal protein fusion product 1	UBA52	chr19p13.1-p12
Vacuolar protein sorting 28 homolog (S. cerevisiae)	VPS28	chr8q24.3
Williams-Beuren syndrome chromosome region 16	WBSCR16	chr7q11.23
WW domain containing oxidoreductase	WWOX	chr16q23.3-q24.1
X antigen family, member 1D///X antigen family, member 1C///X antigen family, member 1E///X antigen family, member 1///X antigen family, member 1B	XAGE1///XAGE1B///XAGE1C///XAGE1D///XAGE1E	chrXp11.22
Zinc finger protein 449	ZNF449	chrXq26.3

### QRT-PCR validation of selected genes expressed in PR versus GR by SSH

**Figure 1:**
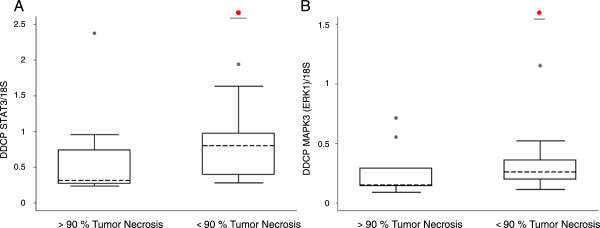
**RTQ-PCR analysis of STAT3 and ERK1 genes. A**: Quantification of STAT3 mRNA with 18S rRNA reference gene transcript confirmed higher STAT3 mRNA levels in poor responder (PR) samples compared with good responder (GR) samples (p = 0.019). **B**: Quantification of MAPK3 (ERK1) mRNA with 18S rRNA reference gene transcript confirmed higher MAPK3 (ERK1) mRNA levels in PR samples compared with GR samples (p = 0.046).

Only *STAT3* mRNA level and *ERK1* mRNA level were significantly different between PR and GR. Quantification of *STAT3* and *ERK1* mRNA transcripts revealed higher mRNA levels in PR compared to GR samples (p = 0.019 and p = 0.046 respectively). The mean level of *STAT3* mRNA was 0.820 [0.280-13.970] in PR versus 0.310 [0.230-2.370] in GR samples (Figure 1A) and the mean level of *ERK1* was 0.270 [0.110-4.340] in PR versus 0.150 [0.088-0.710] in GR samples (Figure 1B).

### Validation at protein level using immunohistochemistry for pSTAT3 and pERK1 (Tables 5 and 6)

**Figure 2:**
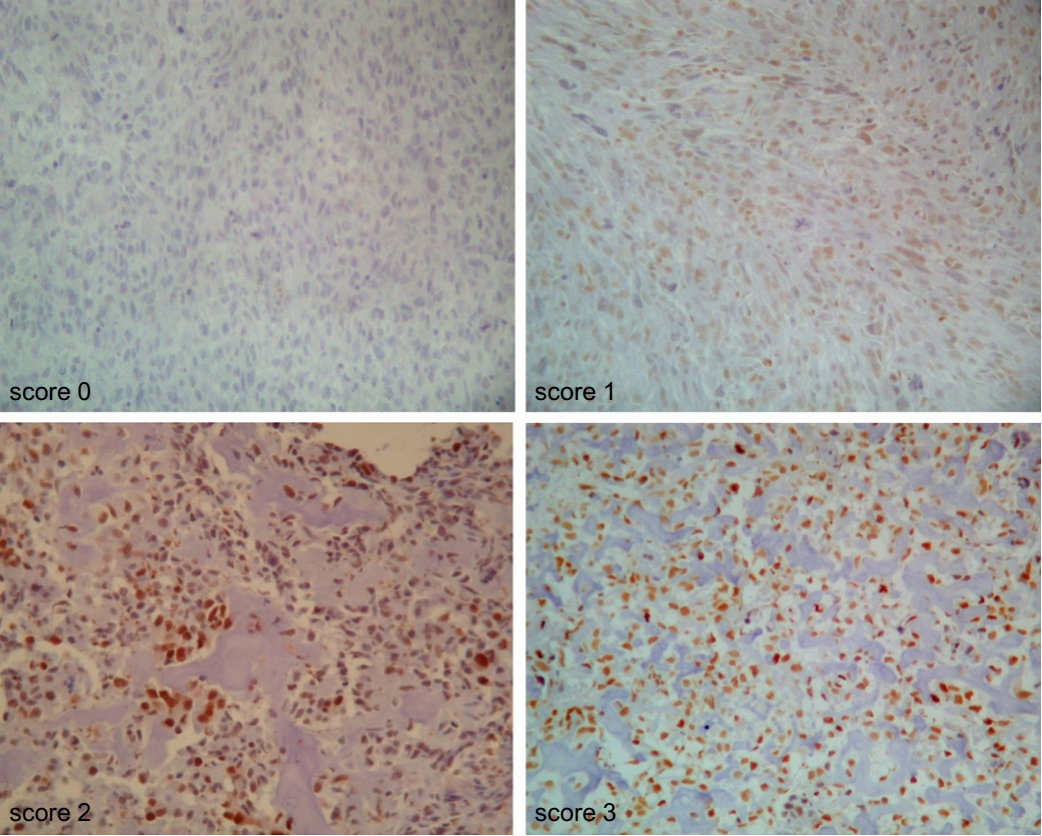
**IHC pSTAT3 scores.** Score 0: negative staining (X200). Score 1: *>50% of nuclei* are labeled with low staining intensity (X200). Score 2: <50% cells with moderate staining intensity and <25% of cells are highly stained (X200). Score 3: more than 50% of nuclei are stained with moderate staining intensity (X200).

pSTAT3 nuclear expression was examined in 45 cases out of 52 and a high score was observed in 20 cases (score 2 and 3). pERK1 expression was examined in 45 cases out of 52 (low score in 25 cases and high score in 20 cases) (Figure 2). pSTAT3 protein expression was correlated to poor response to chemotherapy for a percentage of viable residual cells ≤10%, with the higher scores in the PR group (p = 0.036). A statistically significant correlation was also found between pERK1 protein expression and response to chemotherapy when comparing low scores (0-1) versus high scores (2-3) (p = 0.007). Moreover, the correlation between the expression of pSTAT3 and pERK1 in IHC and the response to chemotherapy remained statistically significant for patients under 25 years (p = 0.024 and p = 0.010 respectively). For a percentage of viable residual cells lower than 5%, a statistically significant correlation was still found between pSTAT3 or pERK1 protein expression and response to chemotherapy (p = 0.013 and p = 0.035 respectively). Whatever the threshold (5 or 10%), positive predictive value (probability of belonging to the group of PR in case of high score) of both pSTAT3 and pERK1 in combination was 91%. Negative predictive value (probability of belonging to the group of GR in case of low score) of both pSTAT3 and pERK1 in combination for a 5 and 10% threshold were 69% and 75% respectively

**Table 5:** **Correlation between phosphorylated STAT3 and ERK1 IHC expression to poor response to chemotherapy for a percentage of viable residual cells ≤10%**

	IHC score	Good responders	Poor responders	p-value
Phosphorylated STAT3	0 or 1	16	9	0.036
	2 or 3	6	14	
VPP_(PR)_ = 14/20 = 70%/VPN_(GR)_ = 16/25 = 64%				
Phosphorylated ERK1	0 or 1	17	8	0.007
	2 or 3	5	15	
VPP_(PR)_ = 15/20 = 75%/VPN_(GR)_ = 17/25 = 68%				
Phosphorylated STAT3 and ERK1	Both 0-1	12	4	0.003
	Intermediate	8	7	
	Both 2-3	1	10	
VPP_(both/PR)_ = 10/11 = 91%/VPN_(both/GR)_ = 12/16 = 75%

**Table 6:** **Correlation between phosphorylated STAT3 and ERK1 IHC expression to poor response to chemotherapy for a percentage of viable residual cells lower than 5%**

	IHC score	Good responders	Poor responders	p-value
Phosphorylated STAT3	0 or 1	13	12	0.013
	2 or 3	3	17	
VPP_(PR)_ = 17/20 = 85%/VPN_(GR)_ = 13/25 = 52%				
Phosphorylated ERK1	0 or 1	13	12	0.035
	2 or 3	4	16	
VPP_(PR)_ = 16/20 = 80%/VPN_(GR)_ = 13/25 = 52%				
Phosphorylated STAT3 and ERK1	Both 0-1	11	5	0.007
	Intermediate	5	10	
	Both 2-3	1	10	
VPP_(both/PR)_ = 10/11 = 91%/VPN_(both/GR)_ = 11/16 = 69%

## Discussion

SSH is a molecular biology technique that enables the identification of differentially expressed genes between two groups with high sensitivity. By comparing PR to GR prior to chemotherapy among patients with an osteosarcoma, we found 126 clones. *ERK1* and *STAT3*, the genes selected on the basis of their roles in tumorigenesis or chemoresistance, were further studied by QRT-PCR in an independent cohort. *ERK1* and *STAT3* expressions assessed by QRT-PCR and IHC were significantly linked to the response to chemotherapy. The protein encoded by *ERK1* is a member of the MAP kinase family and acts in a signalling cascade that regulates various cellular processes such as proliferation, differentiation, and cell cycle progression in response to a variety of extracellular signals. We found ERK1/2 positivity score by IHC and ERK1/2 IHC high score (score 2 and 3) in 78% and 51% of our cohort, respectively. These results suggested that ERK1/2 pathway could be involved in osteosarcoma as it has supported by Pignochino et al. study that showed activated ERK 1/2 pathway in 66.6% of osteosarcoma samples. Moreover, the same team also showed that Sorafenib, a tyrosine kinase inhibitor, blocks tumor growth, angiogenesis and metastatic potential in preclinical models of osteosarcoma through a mechanism potentially involving the inhibition of *ERK1/2* [29]. No attempt to investigate the link between *ERK1* expression and response to chemotherapy was made in vivo. However, our work suggested that *ERK1* could be involved in drug resistance as reported recently by Si et al. with an approach by RNAi-mediated knockdown of *ERK1/2* inhibiting cell proliferation and invasion and increasing chemosensitivity to cisplatin in human osteosarcoma U2-OS cells in vitro [30].

*STAT3* is one of the transcription factors reported to play an important role in tumor survival, proliferation, angiogenesis and metastasis. In normal cells, *STAT3* is activated transiently to maintain homeostasis. However, if *STAT3* continues to be activated, the abnormal level of expression can trigger oncogenic pathways. Aberrant active *STAT3* promotes uncontrolled growth and survival through dysregulation of expression of downstream targeted genes including *survivin*, *Bcl-xL*, *Bcl-2*, *Mcl-1*, *c-Myc* and *cyclin D1*. Constitutive activation of the STAT3 pathway has recently been shown in several malignancies, especially osteosarcoma [31]. It has recently been implicated in resistance to chemotherapy-induced apoptosis [32]. Furthermore, activation of *STAT3* in several cancers has been found to be correlated with clinical outcome especially in osteosarcoma. A high level of expression of STAT3 by IHC in 76 biopsies of patients with an osteosarcoma was a poor prognostic factor for both overall survival and disease-free survival in univariate and multivariate analysis [33]. High staining with pSTAT3 was also of prognostic value in another series of 51 conventional osteosarcomas [34]. In addition, inhibition of *STAT3* plays a role in proliferation, apoptosis and migration in osteosarcoma cells in vitro. The down-regulation of *STAT3* by miR-125b suppresses in vitro proliferation and migration of osteosarcoma cells [35]. *STAT3* inhibition by RNA interference induces inhibition of proliferation and apoptosis enhancement in osteosarcoma cells [33]. The novel curcumin analog FLLL32 decreases *STAT3* DNA binding activity and expression, and induces apoptosis in osteosarcoma cell lines [36]. The small molecules, LLL12 and FLLL32, inhibit *STAT3* phosphorylation and exhibit potent growth suppressive activity in osteosarcoma cells and tumor growth in mice [37]. In contrast, oncostatin M promotes *STAT3* activation, VEGF production, and invasion in osteosarcoma cell lines [38]. Finally, *STAT3* is involved in drug resistance in osteosarcoma cell lines. Ryu et al. recently showed that the STAT3 pathway was overexpressed in MDR osteosarcoma cells and that inhibitors of *STAT3* such as CDDO-Me could reduce resistance to doxorubicin in these cell lines [31]. In our study, we have showed an expression of pSTAT3 in 58% of cases. This activated STAT3 pathway was correlated to poor response to chemotherapy. Thus, our results are consistent with the results of the literature in vitro through the analysis of patient samples.

The effects of *EGFR* are mediated by activation of downstream signal transduction cascades that include Janus tyrosine kinases (*Jak*), Signal Transducers and Activators of Transcription (*STAT*), Phophatidyl Inositol 3 Kinase (*PI3K*)/Akt and Ras/RAf/MAP kinase (*ERK*). The prognostic value of *EGFR* and its downstream signaling molecules such as STAT3 and ERK1 have been studied in many tumor types. Only one study [39] simultaneously examined the status of *EGFR* and four downstream molecules - pSTAT3, pERK1, pAkt, survivin - by IHC in 47 samples of conventional osteosarcomas. *ERK1* and *survivin* expression were statistically correlated with survival. A high expression was negatively correlated with prognosis. Furthermore, *EGFR* expression was correlated with expression of *ERK1* and it was observed a significant association of *survivin* expression with *STAT3* and *ERK* activation. These results and ours support the idea that ERK is a downstream signaling molecule of EGFR and also suggest a link between the EGFR signaling pathway and drug resistance through *ERK1* and *STAT3* expression in conventional osteosarcoma.

## Conclusions

We have shown that high pSTAT3 and pERK1 expression in the biopsies are suggestive of poor response to chemotherapy. The elevated positive predictive value of high score of both pSTAT3 and pERK1 in combination (91%) highly suggests that IHC test could be used at the time of diagnosis to stratifying patients enrolled in randomized trials. Our results also suggest that *STAT3* and *ERK1* inhibitors might be used in combination with common chemotherapeutic drugs in osteosarcoma in order to increase the response to chemotherapy and to improve the prognosis. Finally, other genes identified by SSH remain to be explored and a prospective validation phase on a larger cohort is still needed before these biomarkers could be used in clinical practice.
